# Dental Times

**Published:** 1851-10

**Authors:** A. A. Blandy


*Dental Times.*—We take the liberty to call the attention of our readers to the recent issue of the Times, which has been generally circulated for the purposes and reasons therein set forth. And we beg this opportunity of returning acknowledgments for the flattering reception it has met with from many quarters, even thus early in its existence.

We must also be permitted to mention that an early response to its solicitation for subscription is desirable. We shall endeavor to improve each succeeding number, and give a general summary of all professional miscellanies of common interest.

Its nominal subscription price is so small as to preclude the possibility of pecuniary emolument, but we rely upon a liberal support, both in *subscription* and *contributions,* to enable us to meet our increased outlay and additional labor. A. A. BLANDY.

